# Comment on “Sex Differences in the Association between Night Shift Work and the Risk of Cancers: A Meta-Analysis of 57 Articles”

**DOI:** 10.1155/2019/9263862

**Published:** 2019-05-09

**Authors:** Pengfei Sun, Minglei Bi, Yipeng Su, Zhenyu Chen

**Affiliations:** ^1^Department of Medicine, Qingdao University, 266000 Qingdao, China; ^2^Department of Plastic Surgery, Affiliated Hospital of Qingdao University, 266000 Qingdao, China

We carefully read the published study by Liu et al. about sex differences in the association between night shift work and the risk of cancer [1]. However, we found many problems in the article.

Firstly, according to the requirements of PRISMA [2], detailed retrieval strategies for at least one database should be provided in the article. But the author did not mention it in the article. They only provided keywords in the article instead.

Secondly, the authors stated in their article that they tested heterogeneity between studies using the *I*^2^ statistic with *I*^2^ ≥ 50% indicating heterogeneity, and if no significant heterogeneity existed, a fixed effects model was adopted; otherwise, a random effects model was used. Although their statement was correct, they did not do so. For example, the random effects model should be used for the meta-analysis of (a) women (*I*^2^ = 78.3%) and (b) men (*I*^2^ = 62.3%) while the fixed effects model was used in Figure 2.

Thirdly, in the article, the dose-response analysis is not rigorous. A binary analysis of dose-response relationship should be performed before the dose-response analysis. If there is no statistical difference in the binary analysis of dose-response relationship, there is no need for the dose-response analysis.

Fourthly, publication bias will affect the final results of meta-analysis [3]. However, the article has obvious publication bias (*p* = 0.001) and high heterogeneity, so we think the conclusion of the article is not credible.

In summary, based on the problems in the article, we question the final conclusion of the article.
